# Pretreatment Affects Profits From Xylanase During Enzymatic Saccharification of Corn Stover Through Changing the Interaction Between Lignin and Xylanase Protein

**DOI:** 10.3389/fmicb.2021.754593

**Published:** 2021-12-24

**Authors:** Xiaoting Feng, Yini Yao, Nuo Xu, Hexue Jia, Xuezhi Li, Jian Zhao, Shicheng Chen, Yinbo Qu

**Affiliations:** ^1^State Key Laboratory of Microbial Technology, Shandong University, Qingdao, China; ^2^Department of Clinical and Diagnostic Sciences, School of Health Sciences, Oakland University, Rochester, MI, United States

**Keywords:** pretreatment, enzymatic hydrolysis, xylanase, lignin, adsorption

## Abstract

Effective pretreatment is vital to improve the biomass conversion efficiency, which often requires the addition of xylanase as an accessory enzyme to enhance enzymatic saccharification of corn stover. In this study, we investigated the effect of two sophisticated pretreatment methods including ammonium sulfite (AS) and steam explosion (SE) on the xylanase profits involved in enzymatic hydrolysis of corn stover. We further explored the interactions between lignin and xylanase Xyn10A protein. Our results showed that the conversion rates of glucan and xylan in corn stover by AS pretreatment were higher by Xyn10A supplementation than that by SE pretreatment. Compared with the lignin from SE pretreated corn stover, the lignin from AS pretreated corn stover had a lower Xyn10A initial adsorption velocity (13.56 vs. 10.89 mg g^−1^ min^−1^) and adsorption capacity (49.46 vs. 27.42 mg g^−1^ of lignin) and weakened binding strength (310.6 vs. 215.9 L g^−1^). Our study demonstrated the low absolute zeta potential and strong hydrophilicity of the lignin may partly account for relative weak interaction between xylanase protein and lignin from AS pretreated corn stover. In conclusion, our results suggested that AS pretreatment weakened the inhibition of lignin to enzyme, promoted the enzymatic hydrolysis of corn stover, and decreased the cost of enzyme in bioconversion.

## Introduction

The bioconversion process of lignocellulosic biomass to bioethanol under environmentally friendly, low-energy consumption and mild process conditions has been widely studied in the past decades (Kumar et al., 2008; Bilal et al., 2017). However, the low substrate saccharification efficiency limit its industrial application because of the recalcitrance of native lignocellulose and the high cost of lignocellulose-degrading enzymes (cellulase and hemicellulase) (Berlin et al., 2005). Pretreatment is an indispensable step in the bioconversion of lignocellulosic materials, which efficiently destroyed the tight lignocellulose structure and improved enzymatic digestibility (Tan et al., 2013; Karimi and Taherzadeh, 2016). Several pretreatment methods have been attempted including dilute acid pretreatment, alkali pretreatment, steam explosion (SE) pretreatment, and ionic liquid pretreatment (Zhu and Pan, 2010). The different pretreatments improve the enzymatic digestibility of lignocellulosic materials through different mechanisms involved in the part removal of chemical components (mainly hemicellulose or lignin), increased surface area of substrate, and decreased crystallinity of cellulose (Liu et al., 2017; Ying et al., 2018). Among them, the mechanisms in the SE pretreatment and bisulfite pretreatment are extensively studied because both of them are popular and potential practical pretreatment methods (Rahikainen et al., 2013a; Tan et al., 2015). After the SE and bisulfite pretreatment, a relatively high conversion of cellulose into glucose could be reached with the assistance of enzymatic hydrolysis (Tan et al., 2015; Siddhu et al., 2019). On the other hand, different changes in chemical components of lignocellulose and lignin properties occurred during SE and ammonium sulfite (AS) pretreatment. For example, SE pretreatment hydrolyzed hemicellulose and solubilized a small portion of lignin while causing lignin degradation and condensation, which increased the hydrophobicity of lignin and its adsorption affinity to cellulases (Rahikainen et al., 2013a). During bisulfite pretreatment, hemicellulose is relatively modest degradation, and part of the lignin is converted to lignosulfonate, which increased hydrophilicity of lignin (Wang et al., 2013; Tan et al., 2015).

However, the residue lignin in the pretreated lignocellulose could hinder the enzymatic hydrolysis of lignocellulose to fermentable sugars to act as a physical barrier, causing non-productive adsorption of enzymes (Li et al., 2018; Xu et al., 2019). It has been reported that lignin content is negative related to the enzymatic digestibility of lignocellulose (Dos Santos et al., 2018; Xu et al., 2020); moreover, lignin characteristics affected the non-productive enzyme adsorption onto lignin (Huang et al., 2016). The pretreatment process often changed the residual lignin characteristics; for example, these residual lignins have different molecular weights, structures, and functional groups after different pretreatments. Thus, it is very necessary to investigate the effect of different pretreatment processes on enzymatic hydrolysis and non-productive adsorption of enzyme(s) onto lignin.

Xylanase, as a major hemicellulase component (Li Y. et al., 2015), has been reported to play an important role in enzymatic hydrolysis of hemicellulose and cellulose (Inoue et al., 2015; Miao et al., 2017). However, xylanase can be non-productively adsorbed onto lignin. For example, both the ethanol organosolv dissolved lignin (DL) and the enzymatic residual lignin (ERL) prepared from Douglas-fir exhibited much strong adsorption ability for Multifect xylanase preparation (Berlin et al., 2006). Further, the acid insoluble lignin (AIL) with high hydrophobicity and low zeta potential had higher adsorption capacity toward xylanase than the enzymatic hydrolysis lignin (EHL) did (Chen et al., 2020). In these studies, however, researchers just used the crude xylanase preparations or lignin samples from unpretreated raw materials to simply evaluate the adsorption ability of lignin (mainly total protein decreased). So far, the interactions between lignin from different-methods-pretreated lignocellulose and pure xylanase (e.g., adsorption kinetics and isotherms, and combining features between enzyme and lignin) and their differential interaction mechanism are not clearly understood. The understanding on the adsorption behavior of pure xylanase onto different lignins will contribute to generate “weak-lignin-adsorbed” enzymes. For example, one can modify enzyme structures or change lignin properties to weaken and/or eliminate xylanase absorption to the lignin, which will enhance enzymatic degradation of hemicellulose and reduce cost of enzyme in enzymatic hydrolysis process.

*Penicillium oxalicum* JU-A10-T is a cellulase and hemicellulase producer, and the enzyme from the strain has high commercial potential in the bioconversion of agricultural wastes such as corn stover (CS; Liu et al., 2013). In this study, we first investigated the effects of a purified xylanase (Xyn10A) from the *P. oxalicum* JU-A10-T on the enzymatic hydrolysis efficiency of CS pretreated by SE and AS. We further compared the adsorption isotherms and adsorption kinetics, and the binding stability between the protein and lignin using milled wood lignin (MWL) extracted from SE- and AS-pretreated corn stover and unpretreated corn stover. This study explained the reason why pretreatment methods affect xylanase profits involved in enzymatic hydrolysis of CS from the point of the interactions between lignin and xylanase, and provided a reference for looking for ways to improve the enzymatic hydrolysis efficiency of lignocellulose and decrease enzyme cost.

## Materials and Methods

### Materials

Corn stover was obtained from a local farm in Qingdao, Shandong Province, China. It was cut into ∼3-cm-length segments for further experiments. AS pretreatment of CS was conducted in a rotary electrothermal pressure digester under the conditions of AS dosage of 20% (on dry weight of CS), solid/liquid ratio of 1:5, and 160°C for 30 min. The pretreated solid residues were collected by filtration, rinsed with tap water (till neutral pH was obtained), and stored at 4°C for subsequent experiments. SE-pretreated corn stover was provided by COFCO Shandong Peanut Products Co., Ltd, Qingdao, China. Chemical components of unpretreated and pretreated corn stover were determined according to the protocol established by the National Renewable Energy Laboratory (Sluiter et al., 2011).

Xyn10A (PDE_08094) is the most predominant endo-beta-1,4-xylanase in crude enzyme preparation from *P. oxalicum* JU-A-10T (accounting for 15.21% of total extracellular proteins) (Liu et al., 2013). The Xyn10A was produced in an engineered *P. oxalicum* A11Δ strain (the amylase gene amy15A was deleted) (Lu et al., 2018). The purified Xyn10A (as shown in Supplementary Figure 1) was obtained after running through the HisTrap TMFF column.

The commercial cellulase powder SP was provided by Sino Biotechnology Co., Ltd. (Gansu, China) with a filter paper activity of 150 FPU/g.

### Enzymatic Hydrolysis

Enzymatic hydrolysis of unpretreated and pretreated corn stover was conducted in a 50-ml flask in a constant temperature bath shaker. The cellulosic substrate, commercial cellulase SP, and sodium acetate buffer (0.2 M, pH 4.8) were rotated at 150 rpm at 48°C for 72 h. The substrate consistency was 5%, and cellulase dosage was 5 FPU/g substrate [dry matter (DM)]. To assess the effect of xylanase on enzymatic hydrolysis, Xyn10A at the concentration of 0.26 mg protein/g substrate (DM) was added to the hydrolysis system; the sample without Xyn10A addition was used as the control. At different intervals, 0.2 ml of samples was taken out; the glucose and xylose contents were measured using a high-performance liquid chromatography (HPLC) with Aminex HPX-87P column (Shimadzu, Kyoto, Japan).

The conversions of glucan and xylan were calculated according to Eqs (1, 2), respectively:


(1) $$\text{Glucan}\text{conversion}\left(\%\right)=M_{1}/G\times 0.9\times 100\%$$

(2) $$\text{Xylan}\text{conversion}\left(\%\right)=M_{2}/X\times 0.88\times 100\%$$

where *M*_1_ and *M*_2_ are the glucose and xylose released from hydrolysis, respectively (mg); and *G* and *X* are the theoretical content of cellulose and xylose in substrate, respectively (mg).

### Lignin Preparation

The cellulolytic enzyme lignin (CEL) was prepared according to the method reported by Yoo et al. (2017). Firstly, the CS sample was Soxhlet-extracted with ethanol/toluene (1:2, v/v) for 24 h, and then it was treated for three times using the cellulase (100 FPU/g dry substrate). Subsequently, the solid residue was incubated in 50 mM of phosphate buffer (pH 7.4) containing 1 U/ml of Pronase (Sigma Chemical Company, St. Louis, MO, United States) to remove residual enzyme at 37°C for 24 h; it was next incubated at 90°C for 2 h in order to inactivate the Pronase. The solid residue was extensively washed and freeze-dried and as CEL.

Then CEL was refined by the MWL method described by Yao et al. (2017). Briefly, the CEL is dissolved in a solution of acetic acid–water (9:1, v/v) at a solid–liquid ratio of 1:10, precipitated in 100 ml of deionized water, and centrifuged to separate the solid. The solid was further dissolved with 1,2-dichloroethane-ethanol mixture (2:1, v/v), precipitated in diethyl ether, and centrifuged for recovering solid fraction. After that, the solid was washed with diethyl ether and vacuum dried at 40°C to obtain MWL. The MWL isolated from SE pretreated corn stover (SEPCS), AS pretreated corn stover (ASPCS), and unpretreated corn stover (UPCS) were designated as SE-MWL, AS-MWL, and UP-MWL, respectively.

### The Adsorption of Xyn10A Onto Different Milled Wood Lignins

The adsorption experiment was conducted at 48°C for 24 h. The reaction system contains 0.01 g of lignin (SE-MWL, AS-MWL, or UP-MWL), 0.2 mg of Xyn10A protein, and acetate buffer (pH 4.8, 50 mM), and the total working volume of the system was 1 ml. To investigate the interaction force between lignin and protein, 200 mM of NaCl and 2.5 mg/g lignin of bovine serum albumin (BSA) were added to the reaction system, respectively. After adsorption, the supernatant was collected by centrifugation (13,000 *g*, 10 min) for protein determination as described by Bradford (1976). All experiments were performed in triplicate, and the result shown in the paper was the mean value.

### Adsorption Kinetics and Isotherms

For the kinetic study, adsorption of Xyn10A onto lignin was conducted in 1 ml of reaction system with 0.01 g of lignin in acetate buffer (50 mM, pH 4.8). After adsorption at different times, the supernatant was collected by centrifugation (13,000 *g*, 10 min) for measuring free protein content by Bradford method with BSA (Sigma Aldrich) as the standard (Bradford, 1976).

The adsorption isotherms were performed at 48°C for 24 h. In detail, various concentrations of xylanase (0.2–2 mg/ml) were mixed with 1 ml of suspension containing 0.01 g of lignin in acetate buffer (50 mM, pH 4.8). After incubation, the supernatant was separated as described above, and the protein concentration was measured by Bradford method mentioned above.

### Analysis of Binding Stability of Xyn10A With Lignin

Firstly, the Xyn10A and different MWL samples were incubated in acetate buffer (50 mM, pH 4.8) at 48°C for 24 h, the lignin concentration was 1% (w/v), and total working volume was 1 ml. After adsorption, the solid fraction with adsorbed Xyn10A was separated by centrifugation (13,000 *g*, 10 min) and termed as lignin–Xyn10A complex. To investigate the binding stability of xylanase Xyn10A with lignin, the lignin–Xyn10A complex was washed three times using an equal volume of fresh buffer (50 mM, pH 4.8) at room temperature, and supernatants were obtained by centrifugation (13,000 *g*, 10 min) after each washing to determine free protein content and xylanase activity. The schematic is shown in Supplementary Figure 2.

The adsorption rate of protein and percentage of residual xylanase activity (*P*_*t*_) in the supernatant after adsorption were calculated according to Eq. (3), and the elution rate (*E*_*t*_) of protein in the complex of lignin–protein after each washing was calculated according to Eq. (4):


(3) $$P_{t}\left(\%\right)=A_{t}/C_{0}\times 100\%$$

(4) $$E_{t}\left(\%\right)=M_{t}/A_{t}\times 100\%$$

where *A*_*t*_ is the content of the adsorbed protein or the xylanase activity of the residual protein in supernature after adsorption at different time *t*, and *C*_0_ is the initial protein content or initial xylanase activity before adsorption. *M*_*t*_ is the content of the protein eluted from the complex of “lignin–Xyn10A” prepared at different adsorption time (*t*) after washing with fresh buffer. The protein adsorbed on the lignin was calculated by subtracting the protein content in the supernatant after adsorption from initial protein content before adsorption. The activity of xylanase was assayed using a DNS reagent as described by Li Z. et al. (2015).

### The Physicochemical Properties of Milled Wood Lignins

The lignin was incubated in 50 ml of sodium acetate buffer solutions (0.05 M, pH 4.8) at 48°C, 200 rpm for 30 min, and the lignin concentration was 0.033% (w/w). Then the lignin solution was left to stand for 60 min, and the supernatant was used for measuring zeta potential of lignin sample using a Zeta Potential Analyzer (ZetaPlus) (Lou et al., 2013). All experiments were performed in triplicate with seven readings to ensure experimental repeatability. The surface hydrophobicity of lignin was estimated by measuring the distribution of the hydrophobic dye Rose Bengal in solution and lignin as described previously (Gessner et al., 2000). Briefly, different concentrations of lignin (2–10 g/L) were incubated with a constant concentration of Rose Bengal (40 mg/L) in 50 mM of citrate buffer (pH 4.8) at 48°C, 150 rpm for 2 h, and then the free dye content in the supernatant was determined at 543 nm using a UV–Vis spectrometer (UV-2550, Shimadzu, Kyoto, Japan). The partition quotient (PQ) is calculated from the ratio of the adsorbed dye (*A*_dye_) over the free dye (*F*_dye_) in solution and was plotted against lignin content. The slope in the linear plotting was defined as the surface hydrophobicity of lignin (L/g).

## Results and Discussion

### Effect of Pretreatments on Chemical Compositions of Corn Stover

The contents of cellulose, lignin, and hemicellulose of native CS were 32.66, 22.32, and 19.56%, respectively (Table 1). Compared with the untreated CS, SE pretreatment led to more degradation of hemicellulose, and the hemicellulose contents of pretreated corn stover were reduced from 19.56 to 5.72%. The cellulose contents increased from 32.66 to 45.51%; under the same conditions, lignin contents increased from 22.32 to 26.18%. Previous studies indicated that SE pretreatment effectively degraded hemicellulose with little lignin elimination and resulted in an increase in the lignin content of the pretreated corn stover (Brownell et al., 1986; Rahikainen et al., 2013a). But AS pretreatment exhibited a good delignification performance, and the lignin content significantly decreased from 22.32 to 12.94% after AS pretreatment. However, the contents of cellulose and hemicellulose increased (from 32.66 to 51.11% and from 19.56 to 22.84%, respectively). The partial removal of lignin may weaken the negative effect of lignin on enzymatic hydrolysis and increase the contents of cellulose and hemicellulose in pretreated corn stover after AS pretreatment. Collectively, our observations here were consistent with previous ones (Wang et al., 2018; Xu et al., 2019).

**TABLE 1:** Chemical compositions of corn stover samples with/without pretreatment (% W/W).

Pretreatment	Cellulose	Lignin			Hemicellulose	Ash
		Acid-insoluble	Acid-soluble	Total		
UPCS	32.66 ± 0.13	0.97 ± 0.15	21.35 ± 0.98	22.32	19.56 ± 1.15	1.28 ± 0.05
SEPCS	45.51 ± 2.92	0.72 ± 0.02	25.46 ± 0.60	26.18	5.71 ± 0.28	10.22 ± 0.55
ASPCS	51.11 ± 0.46	1.73 ± 0.01	11.21 ± 0.35	12.94	22.82 ± 0.82	1.57 ± 0.09

*UPCS, unpretreated corn stover; SEPCS, steam explosion-pretreated corn stover; ASPCS, ammonium sulfite-pretreated corn stover.*

### Importance of Xylanase Xyn10A During Enzymatic Hydrolysis of Corn Stover

**FIGURE 1:**
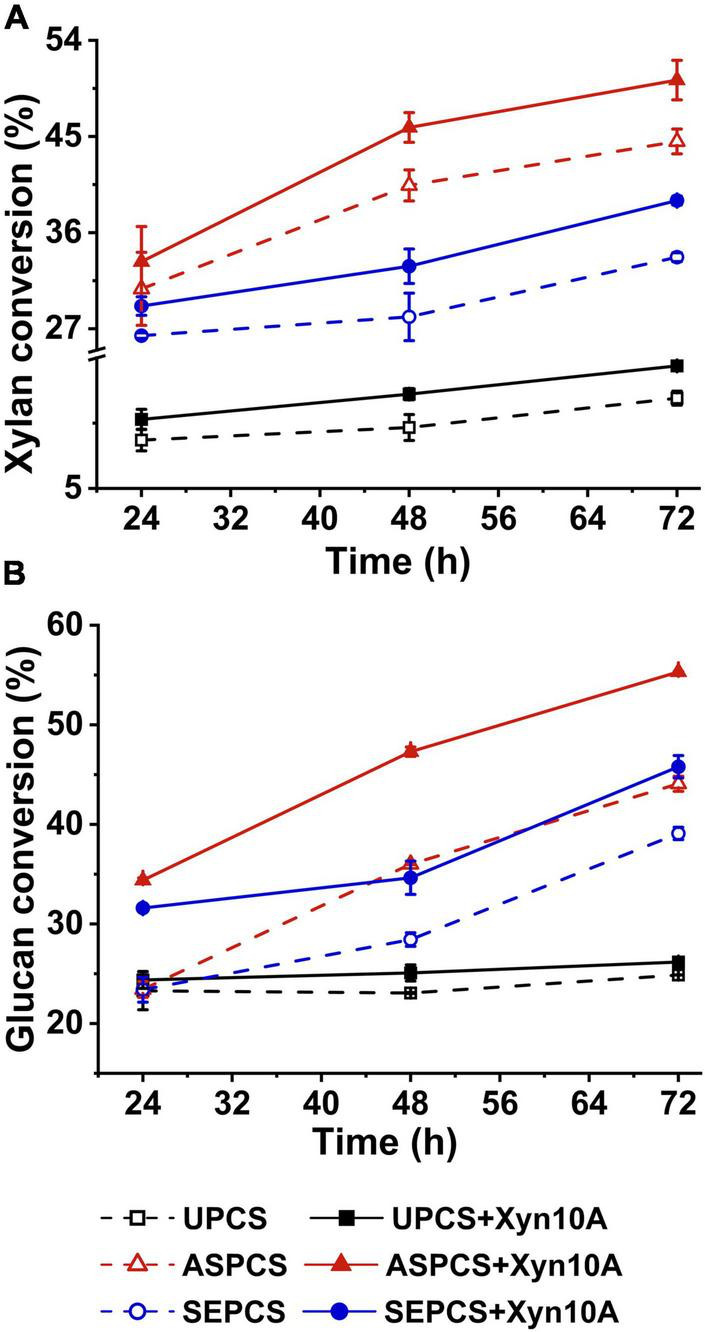
Effect of Xyn10A addition on xylan conversion **(A)** and glucan conversion **(B)** during enzymatic hydrolysis of different-methods-pretreated corn stover. Note: UPCS, unpretreated corn stover; ASPCS, ammonium sulfite pretreated corn stover; SEPCS, steam explosion pretreated corn stover.

Figure 1 illustrates the enzymatic digestibility of pretreated and untreated CS using cellulase SP with/without Xyn10A supplementing as enzyme source. For any one lignocellulosic substrate, the conversions of both glucan and xylan were promoted by adding enzyme Xyn10A to reaction system when compared with that without the addition of Xyn10A (Figures 1A,B). This result indicated that Xyn10A was a necessary component for cellulase system used in enzymatic hydrolysis of the cellulosic substrates to obtain a high conversion of glucan and xylan into fermentable sugars. The enhanced conversion of xylan by adding xylanase to hydrolysis system was reported in previous literatures (Rakotoarivonina et al., 2015; Miao et al., 2018), but the conversion of glucan is also significantly improved by supplementing the pure xylanase Xyn10A (Figure 1B). It is attributed to the synergism between xylanases and cellulases (Inoue et al., 2015). The addition of xylanase into the reaction system during enzymatic hydrolysis resulted in the removal of more hemicellulose in lignocellulosic substrates, which increased the accessibility of cellulase to cellulose.

The conversions of both glucan and xylan of the pretreated corn stover were significantly higher than those of unpretreated substrate (Figure 1), which may be explained by the destruction of the lignocellulose cell structure by pretreatment as well as increase of the accessibility of enzyme to biomass substrate. For example, Tan et al. reported that, after bisulfite pretreatment, specific surface area (both external and internal) of oil palm empty fruit bunch and the pore with larger size increased, which improved the penetration of enzymes into the substrate and the accessibility of enzyme to biomass substrate and led to the improvement of the enzymatic hydrolysis (Tan et al., 2015). Furthermore, it is worth noting that the effect of xylanase supplementing on enzymatic hydrolysis of ASPCS was slightly greater than that of SEPCS (50 and 39% for Xylan conversion, and 55 and 46% for glucan conversion). The possible reasons may account for the above observations: (1) xylan content in the two substrates and distribution of hemicellulose in cell wall were different, leading to the difference on the efficiency of interaction between xylanase and xylan in substrate; for example, more xylan in AS-pretreated corn stover physically hindered the cellulase contacting with cellulose, and xylanase supplementation promotes the hydrolysis of the xylan, thus more effectively improving enzymatic hydrolysis compared with the no adding xylanase. (2) Different pretreatments led to the differences in contents and characteristics of residue lignin in ASPCS and SEPCS (Table 1), affecting non-productive adsorption of enzyme onto lignin.

### Effect of Pretreatments on Interaction Between Lignin With Xyn10A

#### Effect on Adsorption Ability of Lignin to Xylanase

**FIGURE 2:**
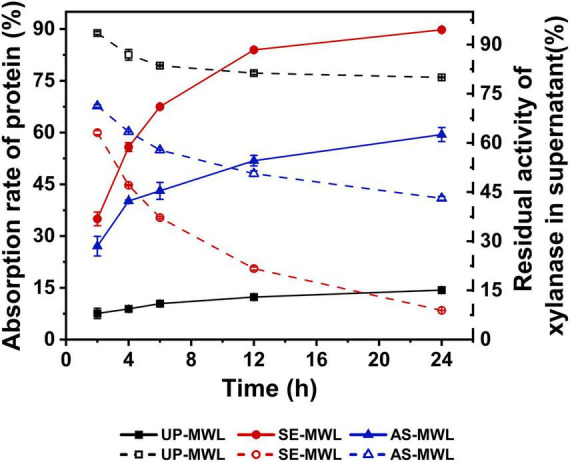
The changes of adsorption rates of Xyn10A during adsorption with different lignins and residual activities of Xyn10A in supernatant after adsorption.

Figure 2 shows the changes of adsorption rates of Xyn10A onto different lignin samples and the changes of residual activities of xylanase in supernature after adsorption over adsorption time.

There was a significantly difference in the adsorption behavior of Xyn10A onto different lignins (Figure 2). Compared with UP-MWL (MWL from unpretreated corn stover), the SE-MWL (MWL from SE-pretreated corn stover) and AS-MWL (MWL from AS-pretreated corn stover) showed higher adsorption rates, indicating that SE and AS pretreatment enhanced the adsorption capacity of lignin. It was unfavorable for enzymatic hydrolysis of lignocellulosic substrate. During the 24-h adsorption process, with the increase of time, the adsorption rates of three lignin increased. At 24 h, about 90% of protein in enzyme solution was adsorbed onto SE-MWL, significantly higher than AS-MWL (about 60%), indicating that the SE-MWL has greater adsorption capacity for Xyn10A than AS-MWL. Similar to the change of protein in the supernatant, the residual activity of xylanase in the supernatant by SE-MWL adsorption was the lowest when compared with that by AS-MWL and UP-MWL, showing that SE-MWL had greater effect on enzyme activity. The difference in the adsorption behaviors of SE-MWL and AS-MWL may be due to the different characteristics of the two lignin samples from different pretreatment processes.

#### Interaction Forces Between Xyn10A and Lignin

**FIGURE 3:**
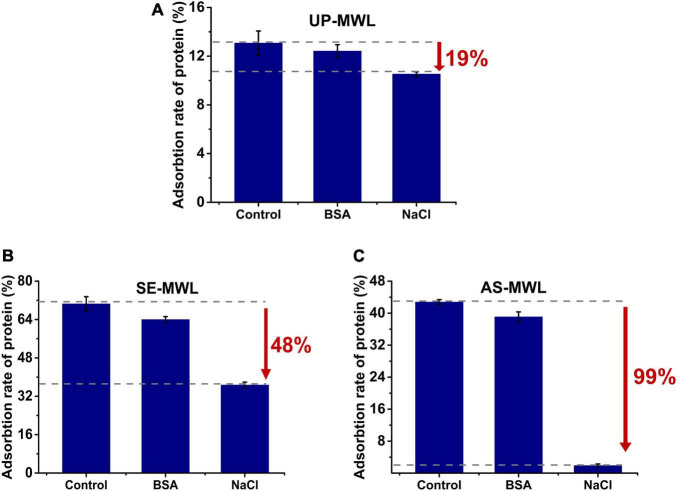
Effect of adding NaCl or BSA to reaction system on the adsorption of Xyn10A onto different lignin samples, i.e. UP-MWL **(A)**, SE-MWL **(B)**, AS-MWL **(C)**, in which control is the sample without addition of NaCl and BSA. BSA, bovine serum albumin.

It was reported that BSA and NaCl were competitively bound to lignin with enzyme by hydrophobic force and electrostatic interaction, respectively (Yang and Wyman, 2006; Lu X. Q. et al., 2017). To investigate the interaction forces between Xyn10A and MWLs, NaCl, and BSA were, respectively, added to adsorption system and their effect on adsorption of Xyn10A onto different lignins (shown in Figure 3).

The addition of both BSA and NaCl reduced the adsorption capacity of lignin (Figure 3). For all the lignin samples, both hydrophobic interaction and electrostatic interaction drove the adsorption of Xyn10A onto lignin. The addition of NaCl resulted in more decrease in adsorption rate of protein than addition of BSA, indicating that electrostatic interaction was relatively the main force for the non-productive adsorption of Xyn10A to the lignin samples, especially for AS-MWL (decreased by 99%, Figure 3C). Compared with UP-MWL, we observed that there was a bigger drop-in adsorption rate of protein after adding NaCl. The AS and SE pretreatment enhanced the electrostatic interaction between Xyn10A and lignin due to the differences of lignin characteristics.

#### Adsorption Kinetics

The pseudo-first-order and pseudo-second-order kinetic models were used for the study of adsorption kinetics.

The pseudo-first-order kinetic model equation:


(5) $$\ln\,\left(\mathrm{Qe}-\mathrm{Qt}\right)=\ln\,\mathrm{Qe}-K_{1}\,t$$

The pseudo-second-order kinetic model equation:


(6) $$t/Q_{t}=1/\left(K_{2}\cdot Q_{e}^{2}\right)+1/Q_{e}\cdot t$$

The initial adsorption velocity *H* (*mg*g^−1^*min*^−1^) was calculated from *K*_2_ and *Q*_*e*_ by the following equation:


(7) $$H=K_{2}\cdot Q_{e}^{2}$$

where *Q*_*e*_(*mg*g^−1^) is the adsorption capacity at equilibrium, *Q*_*t*_ (*mg*g^−1^) is the adsorption capacity at time *t*, *t* is the adsorption time, and *K*_1_(min^−1^) and *K*_2_ (g*mg*^−1^*min*^−1^) are adsorption rate constants of pseudo-first-order and pseudo second-order kinetics, respectively.

**FIGURE 4:**
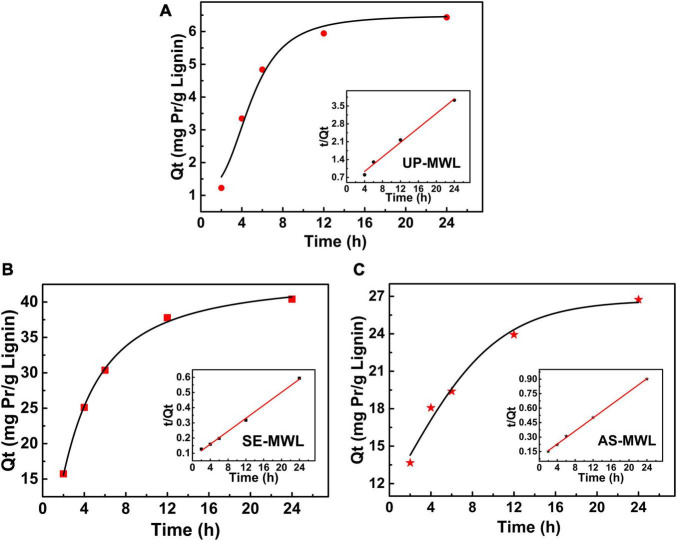
The pseudo-second-order kinetic model of adsorption of Xyn10A onto lignins from unpretreated **(A)** and different pretreated corn stover by SE **(B)** or AS **(C)**. AS, ammonium sulfite; SE, steam explosion.

The fitting curves of the experimental data to pseudo-second-order kinetic model are shown in Figure 4, and the various parameters obtained by the models are listed in Table 2. The adsorbed protein amounts increased rapidly with the increase of adsorption time and then reached the adsorption equilibrium (Figure 4). The adsorption process of Xyn10A onto lignin conformed to the pseudo-second-order kinetic model because the correlation coefficient *R*^2^ was closest to 1. There was different initial adsorption velocity (*H*) and different adsorption capacity (*Q*_*e*_) for Xyn10A onto three lignin samples, and the values of *H* and *Q*_*e*_ for the lignin isolated from SEPCS were greater than those from ASPCS, and the *Q*_*e*_ value was the smallest for lignin from unpretreated corn stover. The results suggested that lignin from SEPCS has stronger and faster adsorption capacity than that from ASPCS and unpretreated corn stover, which could also be seen from the change curves of *t*/*Q*_*t*_ over time.

**TABLE 2:** Adsorption kinetics model parameters.

Kinetic model	Parameters	UP-MWL	SE-MWL	AS-MWL
Pseudo-first-order model	*Q*_*e*_ (*mg*g^−1^)	21.98	26.00	28.15
	*K*_1_ (min^−1^)	32.96	1.380	2.278
	*R* ^2^	0.955	0.882	0.986
Pseudo-second-order model	*Q*_*e*_ (*mg*g^−1^)	7.035	46.75	29.50
	*K*_2_ (g*mg*^−1^*min*^−1^)	0.055	0.006	0.013
	*H*(*mg*g^−1^*min*^−1^)	2.279	13.56	10.89
	*R* ^2^	0.989	0.996	0.999

*UP-MWL, milled wood lignin isolated from unpretreated corn stover; SE-MWL, milled wood lignin isolated from steam explosion-pretreated corn stover; AS-MWL, milled wood lignin isolated from ammonium sulfite-pretreated corn stover.*

#### Adsorption Isotherms

Adsorption isotherms were indispensable for understanding the adsorption characteristics. In this study, five related adsorption isotherm models, i.e., Langmuir model, Hill model, Dubinin–Radushkevich (D-R) model, Freundlich model, and Temkin isotherm model, were constructed to compare the binding affinity of MWLs from different pretreated corn stover with Xyn10A.

The Langmuir adsorption isotherm model equation is shown as follows:


(8) $$C_{e}/Q_{e}=C_{e}/Q_{m,L}+1/\left(Q_{m,L}\cdot K_{L}\right)$$

(9) $$K_{p}=Q_{m,L}\cdot K_{L}$$

The Hill adsorption isotherm model equation is shown as follows:


(10) $$Q_{e}=Q_{m,H}\cdot C_{e}^{n}/\left(K_{\mathrm{ad}}^{{\mathrm{app}}^{-1}}+C_{e}^{n}\right)$$

(11) $$K_{d}={\left(K_{\mathrm{ad}}^{{\mathrm{app}}^{-1}}\right)}^{1/n}$$

The D-R adsorption isotherm model equation is shown as follows:


(12) $$\ln\,Q_{e}=\ln\,Q_{m,D-R}-\beta \,\varepsilon^{2}$$

(13) $$\varepsilon =\mathrm{RT}\cdot \ln\,\left(1+1/C_{e}\right)$$

(14) $$E=1/\sqrt{2\,\beta }$$

The Temkin adsorption isotherm model of commonly used equation is as follows:


(15) $$Q_{e}=B_{1}\,\text{ln}\,A+B_{1}\,\text{ln}\,C_{e}$$

The Freundlich adsorption isotherm equation is shown as follows:


(16) $$\ln\,Q_{e}=\ln\,K_{F}+1/n\cdot \ln\,C_{e}$$

where *Q*_*e*_ (*mg*g^−1^) and *C*_*e*_ (*mg*L^−1^) are adsorption capacity and concentration of Xyn10A in the supernatant at equilibrium, respectively. *Q*_*m*,*L*_ (*mg*g^−1^), *Q*_*m*,*H*_(*mg*g^−1^), and *Q*_*m*,*D*−*R*_(*mg*g^−1^) are the maximum adsorption capacity of the Langmuir, Hill, and D-R adsorption isotherm models, respectively. *K*_*L*_ (L*mg*^−1^) and *K*_*p*_(Lg^−1^) are adsorption affinity and binding strength of the Langmuir model, respectively. $K_{a\,d}^{a\,p\,p^{-1}}$, *n*, and *K*_*d*_ are the apparent association constant, cooperative parameter, and dissociation constant of the Hill model, respectively. β(mol^2^*KJ*^−2^) and ε are the adsorption energy and Polanyi adsorption potential energy of the D-R model, respectively. *R* is the gas constant 8.314 (J*mol*^−1^K^−1^); *T* (K) is the thermodynamic temperature, and *E*(*KJmol*^−1^) is the average free energy. *A* (*mg*L^−1^) and *B* are the equilibrium constant and adsorption heat of the Temkin model, respectively. *K*_*F*_ (Lg^−1^) is the adsorption capacity of the Freundlich model.

The parameters obtained by the five adsorption isotherm models are summarized in Table 3. The correlation coefficient *R*^2^ of the Langmuir, Hill, and D-R isotherm models were much higher than that of the Freundlich and Temkin models, and the *Q*_*e*_ values calculated by the three models were almost consistent with those of the experimental values. It was illustrated that the three models were more suitable for simulating the adsorption process of Xyn10A onto the lignin samples from different pretreated corn stover, which was similar to cellobiohydrolase (CBH; Lu X. et al., 2017).

**TABLE 3:** Parameters from different adsorption isotherm models.

Isotherm model	Parameters	UP-MWL	SE-MWL	AS-MWL
Langmuir	*K*_*p*_(Lg^−1^)	40.76	310.6	215.9
	*K*_*L*_ (L^−1^*mg*)	3.397	6.280	7.877
	*Q*_*m*,*L*_(*mg*g^−1^)	12.00	49.46	27.42
	*R* ^2^	0.993	0.997	0.999
Hill	*Q*_*m*,*H*_(*mg*g^−1^)	10.71	52.59	29.96
	*n*	0.565	1.623	0.776
	*K*_*d*_	0.333	0.236	0.263
	*R* ^2^	0.991	0.980	0.988
Dubinin–Radushkevich	*Q*_*m*,*D*−*R*_ (*mg*g^−1^)	12.09	56.53	25.33
	β(mol^2^*KJ*^−2^)×10^8^	6.100	4.353	1.657
	*E*(*KJmol*^−1^)×10^3^	2.862	3.389	5.439
	*R* ^2^	0.987	0.983	0.986
Freundlich	*K*_*F*_(L*mg*^−1^)	9.365	47.42	23.81
	1/*n*	0.681	0.567	0.279
	*R* ^2^	0.747	0.799	0.920
Temkin	*A*	12.01	22.38	146.6
	*B*	9.266	14.97	4.727
	*R* ^2^	0.940	0.947	0.935

*UP-MWL, milled wood lignin isolated from unpretreated corn stover; SE-MWL, milled wood lignin isolated from steam explosion-pretreated corn stover; AS-MWL, milled wood lignin isolated from ammonium sulfite-pretreated corn stover.*

**FIGURE 5:**
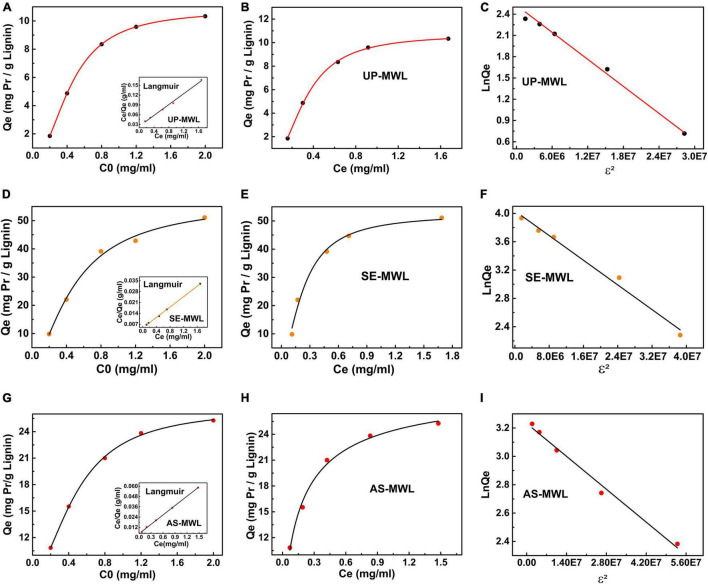
Adsorption isotherm model of Langmuir **(A,D,G)**, Hill **(B,E,H)**, and Dubinin–Radushkevich **(C,F,I)** for the adsorption of Xyn10A onto lignin isolated from AS- and SE-pretreated corn stover and unpretreated corn stover. AS, ammonium sulfite; SE, steam explosion.

The fitting curve of three adsorption isotherm models is shown in Figure 5. There are significant differences on adsorption capacity of Xyn10A onto SE-MWL, AS-MWL, and UP-MWL. Among them, SE-MWL had the highest adsorption capacity for Xyn10A (49.46 mg g^–1^), and then AS-MWL (27.42 mg g^–1^) and UP-MWL were the lowest (12.00 mg g^–1^). This may be due to the acidic environment during SE pretreatment. Yuan et al. reported that the maximum enzyme adsorption capacity of CEL isolated from the dilute alkali pretreated corn stover was lower than that of CEL isolated from the dilute acid pretreated corn stover (Yuan et al., 2018). Interestingly, the effect of pretreatment methods on adsorption affinity of AS-MWL and SE-MWL for Xyn10A (AS-MWL > SE-MWL) was not consistent with the effect on maximum adsorption capacity of the two lignin (SE-MWL > AS-MWL) (Table 3). As the maximum adsorption capacity and adsorption affinity cannot accurately reflect the adsorption characteristics of enzyme onto lignin, the binding strength was introduced to estimate the comprehensive effects of lignin on enzyme (Ying et al., 2018). In general, the higher the binding strength, the more chance to adsorb enzymes (Jiang et al., 2019). The binding strength constant *K*_*P*_ of SE-MWL was the highest (310.6 L g^–1^), about 1.4- and 7.7-fold higher than that of AS-MWL (215.9 L g^–1^) and UP-MWL (40.76 L g^–1^), respectively. These results were consistent with the change trends of adsorption capacity of the different lignin samples (Table 3).

The dissociation constant *K*_*d*_ of Hill model represented the free protein concentration in the supernatant affording 1/2*Q*_*m*,*H*_ (Sugimoto et al., 2012). The value of *K*_*d*_ for SE-MWL was apparently lower than that for AS-MWL and UP-MWL, indicating that Xyn10A could be more quickly bound to the surface of SE-WML compared with the AS-MWL and the UP-MWL (Table 3). n is the cooperative parameter, *n* < 1 presents negatively cooperative adsorption, and *n* > 1 stands for positively cooperative adsorption and is substrate dependent (Sugimoto et al., 2012). The *n* > 1 for SE-MWL showed that the adsorption process of enzyme Xyn10A onto the SE-MWL conformed to positively cooperative adsorption and is substrate dependent, which was interpreted as the phenomenon that the first bound protein induced a conformational change of neighboring binding site, thus having more binding affinity to the second protein molecule. In contrast, *n* < 1 for AS-MWL and UP-MWL, suggesting that adsorption of Xyn10A onto the two lignins was the negatively cooperative adsorption. Furthermore, n also presented the numbers of enzyme molecule occupied by a single lignin molecule surface (Lu X. Q. et al., 2017); the higher *n* value indicates that there was lower steric exclusion among protein molecules (Sugimoto et al., 2012). For SE-MWL, AS-MWL, and UP-MWL, the *n* values were 1.623, 0.776, and 0.565, respectively, indicating that more Xyn10A protein was bound on the surface of SE-MWL lignin molecule. This may be caused by the lower steric exclusion between Xyn10A enzyme molecules. However, the higher steric repulsion between Xyn10A molecules on surface of AS-MWL led to lower capability to attach on the lignin surface, which was consistent with the above result that the AS-MWL had lower adsorption capacity for Xyn10A than SE-MWL.

The D-R model was generally used to evaluate the nature of the interaction between the protein and the binding site (Onyango et al., 2004), which mainly depended on the magnitude of parameter E (average free energy). When the value of E was between 8 and 16, the adsorption process of Xyn10A onto lignin proceeded by chemical adsorption; if E was less than 8, then the adsorption process was controlled by physical adsorption. In this study, the E for all the lignin samples was less than 8, indicating that for all the adsorption processes of Xyn10A onto the different lignin samples were controlled by physical adsorption.

In conclusion, the adsorption isotherms between xylanase and different lignins, the Langmuir, Hill, and D-R isotherm models could be used for exploring and comparing the adsorption behavior of xylanase Xyn10A onto lignin from SE- and AS-pretreated corn stover and unpretreated corn stover. After AS and SE pretreatment, residual lignin in CS, AS-MWL, and SE-MWL showed different adsorption characteristics for xylanase compared with UP-MWL. Compared with the AS-MWL, the SE-MWL showed more and faster binding capacity with Xyn10A molecule because of higher binding strength with Xyn10A and lower steric exclusion between Xyn10A enzyme molecules. The adsorption process of Xyn10A onto the SE-MWL was positively cooperative adsorption and substrate dependent, but it was negatively cooperative and substrate independent for the adsorption process of AS-MWL. However, all the adsorption processes of Xyn10A onto lignin, whether SE-MWL or AS-MWL, were proceeded by physical adsorption.

### Effect of Pretreatments on Binding Stability Between Lignin and Enzyme

Several studies showed that the interaction between enzyme and lignin was completely reversible, and enzyme could be desorbed from substrate by dilution (Kumar and Wyman, 2009; Djajadi et al., 2018). The desorption of enzyme from substrate was beneficial for the recycling of enzyme during hydrolysis. We checked the binding stability of lignin–protein complex formed by the adsorption of enzyme Xyn10A onto lignin samples from different pretreated corn stover using a washing method.

With the increase of adsorption time, the elution rates decreased after the first washing, which showed that the combination of protein with lignin became more and more stable (Table 4). For example, for UP-MWL, AS-MWL, and SE-MWL, when adsorption time was 2 h, 81.15, 19.25, and 16.46% of protein could be eluted from “lignin–enzyme complex” after the first washing, respectively. But they were drastically reduced to 15.20, 5.07, and 2.30%, respectively, after adsorption of 24 h. These results illustrated that after the adsorption for 24 h, almost no protein was released from the “lignin–enzyme complex” during the second and third washing. Kumar and Wyman (2009) reported that after adsorption reached equilibrium, when the buffer containing unbound proteins was replaced by an equal amount of fresh buffer, the desorption of enzyme from lignin from various pretreated poplar was detected (Kumar and Wyman, 2009). But our results show that the just very limited protein-adsorbed-by-lignin may be released by fresh buffer washing (24 h of adsorption time as shown in Table 4). The possible reasons for the differences may be as follows: in Kumar’s research, a commercial mixed enzyme (Spezyme CP cellulase) containing multiple enzyme components was used, but only pure Xyn10A was used in our study. The binding force between enzyme molecules and lignin varies with the types of enzyme molecules; after adsorption, when eluted with fresh buffer, some proteins with weak binding force could be desorbed easily. On the other hand, the adsorption and desorption experiments were conducted under low temperature conditions (4°C) in Kumar’s study, and it was proved that temperature influenced the adsorption and desorption behaviors of enzymes onto lignin (Rahikainen et al., 2013b; Lu X. et al., 2017). Furthermore, Kumar and Wyman (2009) used the Enzyme lignin (EnzL), which contains some carbohydrates and proteins, but in our research, MWL was prepared. The structure of the isolated lignin differs with the different separation methods, different sources of lignin, and enzymes that retain unique characteristics that render different interactions between lignin and enzymes (Li and Zheng, 2017). Our research showing that the “lignin–enzyme complex” formed by adsorption of Xyn10A onto lignin was relatively stable and difficult to be disintegrated to release free xylanase molecule during enzymatic hydrolysis.

**TABLE 4:** The elution rate* of protein adsorbed in the lignin after different washing of lignin–Xyn10A complex by buffer.

Time of adsorption (h)	First washing (%)			Second washing (%)			Third washing (%)		
	UP-MWL	SE-MWL	AS-MWL	UP-MWL	SE-MWL	AS-MWL	UP-MWL	SE-MWL	AS-MWL
2	81.15	16.46	19.25	9.61	3.12	4.63	9.11	2.08	2.79
4	59.09	7.66	12.05	7.04	1.08	1.70	5.02	1.16	1.43
6	40.21	6.05	10.06	4.11	0.77	1.37	3.16	0.59	0.83
12	26.78	4.32	6.92	1.95	0.36	0.82	1.60	0.27	0.45
24	15.02	2.30	5.07	ND**	ND	ND	ND	ND	ND

*UP-MWL, milled wood lignin isolated from unpretreated corn stover; SE-MWL, milled wood lignin isolated from steam explosion-pretreated corn stover; AS-MWL, milled wood lignin isolated from ammonium sulfite-pretreated corn stover.*

*Based on protein content of adsorbed protein in complex of lignin–enzyme.

**ND: not detected.

Furthermore, the Xyn10A protein adsorbed onto SE-MWL was more difficult to be desorbed than that onto AS-MWL, indicating that the “lignin–enzyme complex” formed by adsorption of Xyn10A onto SE-MWL was more stable. It was previously reported that irreversible binding of cellulase to lignin varied with pretreatment types (Kumar and Wyman, 2009). The adsorption isotherms showed that SE-MWL had higher binding strength with Xyn10A than AS-MWL did. It seemed that the “lignin–enzyme complex” formed by SE-MWL had higher stability than AS-MWL. The stable “lignin–enzyme” complex formed by the SE-MWL adsorption was hard to be dissociated to free enzyme molecules by washing, thus hindering enzymatic hydrolysis of lignocellulose.

### Effect of Pretreatments on Lignin Characteristics

Due to many active groups in the lignin structure, the physicochemical properties and structure of lignin were usually changed during chemical pretreatment (Kellock et al., 2019; Xu et al., 2019). The lignin characteristics could significantly affect the non-productive adsorption of enzymes onto lignin (Nakagame et al., 2011; Xu et al., 2020). The hydrophobic interaction and electrostatic interaction were the main driving forces for the adsorption of Xyn10A onto the lignin as in the above discussion. Therefore, the surface charge and hydrophobicity of different lignin samples were characterized (Table 5).

**TABLE 5:** The zeta potential and hydrophobicity of lignin samples.

Properties	UP-MWL	SE-MWL	AS-MWL
Zeta potential (*mv*)	−9.66 ± 0.35	−34.75 ± 0.31	−14.83 ± 1.06
Hydrophobicity (*L*/*g*)	0.3961	0.6097	0.5048

*UP-MWL, milled wood lignin isolated from unpretreated corn stover; SE-MWL, milled wood lignin isolated from steam explosion-pretreated corn stover; AS-MWL, milled wood lignin isolated from ammonium sulfite-pretreated corn stover.*

The zeta potential values of three MWLs were −9.66 mV for UP-MWL, −14.83 mV for AS-MWL, and −34.75 mV for SE-MWL. SE-MWL and AS-MWL exhibited 3.6 times and 1.5 times higher negative surface charges than UP-MWL did. As Xyn10A enzyme molecule was positive under the experiment conditions, the higher negative zeta potential caused stronger electrostatic attraction between lignin and Xyn10A molecules. This might partly explain why SE-MWL had higher adsorption ability and binding strength with Xyn10A than AS-MWL and UP-MWL did.

Many studies reported that lignin with greater hydrophobicity exhibited a stronger inhibitory effect on enzyme activities (Li et al., 2016; Kellock et al., 2019). The hydrophobicity of SE-MWL was 0.6097 L/g, which was also higher than that of AS-MWL (0.5048 L/g) and UP-MWL (0.3961 L/g) (Table 5). The higher surface hydrophobicity of lignin enhanced the hydrophobic binding between lignin with Xyn10A molecule. Table 3 also indicates that there was higher binding strength (high *K*_*p*_ value) and higher adsorption affinity (high *Q*_*m*_ value) between the SE-MWL with higher hydrophobicity and Xyn10A. Previous studies reported that the hydrophobicity of lignin was positively correlated with its inhibitory effect on the enzymatic hydrolysis efficiency of cellulase (Huang et al., 2019; Lin et al., 2019). Compared with the SE-MWL, lower electrostatic attraction and hydrophobic interaction between AS-MWL with Xyn10A weakened the unproductive adsorption of Xyn10A onto the lignin, which was in favor of enzymatic hydrolysis.

## Conclusion

In summary, xylanase was important for enzymatic hydrolysis of CS pretreated by SE and AS, especially AS pretreated corn stover. However, pretreatment methods affected the action efficiency and absorption behaviors of xylanase in the enzymatic hydrolysis. Compared with SE pretreatment, AS pretreatment gave lower absolute zeta potential and stronger hydrophilicity in residual lignin, which led to lower initial adsorption velocity and lower adsorption capacity of xylanase onto residual lignin. AS such, lower binding strength between lignin and Xyn10A and the weak interaction between xylanase and lignin after AS pretreatment was beneficial to the action of xylanase on substrate, thus enhancing enzymatic hydrolysis.

## Data Availability Statement

The raw data supporting the conclusions of this article will be made available by the authors, without undue reservation.

## Author Contributions

XF conducted most of the experiments and data analysis and drafted the manuscript. XF, YY, and NX participated in parts of the experiments. HJ, YQ, and SC revised the manuscript. JZ and XL conceived the study and provided helpful suggestions and edits for the manuscript. All authors contributed to the article and approved the submitted version.

## Conflict of Interest

The authors declare that the research was conducted in the absence of any commercial or financial relationships that could be construed as a potential conflict of interest.

## Publisher’s Note

All claims expressed in this article are solely those of the authors and do not necessarily represent those of their affiliated organizations, or those of the publisher, the editors and the reviewers. Any product that may be evaluated in this article, or claim that may be made by its manufacturer, is not guaranteed or endorsed by the publisher.

## References

[B1] Berlin A. Balakshin M. Gilkes N. Kadla J. Maximenko V. Kubo S. (2006). Inhibition of cellulase, xylanase and beta-glucosidase activities by softwood lignin preparations. *J. Biotechnol*. 125 198–209. 10.1016/j.jbiotec.2006.02.021 16621087

[B2] Berlin A. Gilkes N. Kurabi A. Bura R. Tu M. Kilburn D. (2005). Weak lignin-binding enzymes: a novel approach to improve activity of cellulases for hydrolysis of lignocellulosics. *Appl. Biochem. Biotechnol.* 121-124 163–170. 10.1385/abab:121:1-3:0163 15917596

[B3] Bilal M. Asgher M. Iqbal H. M. Hu H. Zhang X. (2017). Biotransformation of lignocellulosic materials into value-added products-A review. *Int. J. Biol. Macromol*. 98 447–458. 10.1016/j.ijbiomac.2017.01.133 28163129

[B4] Bradford M. M. (1976). A rapid and sensitive method for the quantitation of microgram quantities of protein utilizing the principle of protein-dye binding. *Anal. Biochem*. 72 248–254. 10.1016/0003-2697(76)90527-3 942051

[B5] Brownell H. H. Yu E. K. Saddler J. N. (1986). Steam-explosion pretreatment of wood: effect of chip size, acid, moisture content and pressure drop. *Biotechnol. Bioeng*. 28 792–801. 10.1002/bit.260280604 18555395

[B6] Chen X. Xin D. L. Sun F. F. Zhang J. H. (2020). Factors affecting the hydrolytic action of xylanase during pennisetum saccharification: role of lignin. *Cellulose* 27 3143–3152. 10.1007/s10570-020-02996-z

[B7] Djajadi D. T. Pihlajaniemi V. Rahikainen J. Kruus K. Meyer A. S. (2018). Cellulases adsorb reversibly on biomass lignin. *Biotechnol. Bioeng*. 115 2869–2880. 10.1002/bit.26820 30132790 PMC6282830

[B8] Dos Santos A. C. Ximenes E. Kim Y. Ladisch M. R. (2018). Lignin-enzyme interactions in the hydrolysis of lignocellulosic biomass. *Trends. Biotechnol.* 37 518–531. 10.1016/j.tibtech.2018.10.010 30477739

[B9] Gessner A. Waicz R. Lieske A. Paulke B. Mader K. Muller R. H. (2000). Nanoparticles with decreasing surface hydrophobicities: influence on plasma protein adsorption. *Int. J. Pharm*. 196 245–249. 10.1016/S0378-5173(99)00432-9 10699728

[B10] Huang C. He J. Min D. Lai C. Yong Q. (2016). Understanding the nonproductive enzyme adsorption and physicochemical properties of residual lignins in moso bamboo pretreated with sulfuric acid and kraft pulping. *Appl. Biochem. Biotechnol*. 180 1508–1523. 10.1007/s12010-016-2183-8 27380421

[B11] Huang C. Lin W. Lai C. Li X. Jin Y. Yong Q. (2019). Coupling the post-extraction process to remove residual lignin and alter the recalcitrant structures for improving the enzymatic digestibility of acid-pretreated bamboo residues. *Bioresour. Technol*. 285:121355. 10.1016/j.biortech.2019.121355 31004950

[B12] Inoue H. Kishishita S. Kumagai A. Kataoka M. Fujii T. Ishikawa K. (2015). Contribution of a family 1 carbohydrate-binding module in thermostable glycoside hydrolase 10 xylanase from *Talaromyces cellulolyticus* toward synergistic enzymatic hydrolysis of lignocellulose. *Biotechnol. Biofuels* 8:77. 10.1186/s13068-015-0259-2 26000036 PMC4440266

[B13] Jiang C. Wang X. Qin D. Da W. Hou B. Hao C. (2019). Construction of magnetic lignin-based adsorbent and its adsorption properties for dyes. *J. Hazard. Mater*. 369 50–61. 10.1016/j.jhazmat.2019.02.021 30772687

[B14] Karimi K. Taherzadeh M. J. (2016). A critical review on analysis in pretreatment of lignocelluloses: degree of polymerization, adsorption/desorption, and accessibility. *Bioresour. Technol*. 203 348–356. 10.1016/j.biortech.2015.12.035 26778166

[B15] Kellock M. Maaheimo H. Marjamaa K. Rahikainen J. Zhang H. Holopainen-Mantila U. (2019). Effect of hydrothermal pretreatment severity on lignin inhibition in enzymatic hydrolysis. *Bioresour. Technol*. 280 303–312. 10.1016/j.biortech.2019.02.051 30776657

[B16] Kumar R. Singh S. Singh O. V. (2008). Bioconversion of lignocellulosic biomass: biochemical and molecular perspectives. *J. Ind. Microbiol. Biotechnol*. 35 377–391. 10.1007/s10295-008-0327-8 18338189

[B17] Kumar R. Wyman C. E. (2009). Access of cellulase to cellulose and lignin for poplar solids produced by leading pretreatment technologies. *Biotechnol. Prog*. 25 807–819. 10.1002/btpr.153 19504581

[B18] Li J. Lu M. Guo X. Zhang H. Li Y. Han L. (2018). Insights into the improvement of alkaline hydrogen peroxide (AHP) pretreatment on the enzymatic hydrolysis of corn stover: chemical and microstructural analyses. *Bioresour. Technol*. 265 1–7. 10.1016/j.biortech.2018.05.082 29860078

[B19] Li X. Zheng Y. (2017). Lignin-enzyme interaction: mechanism, mitigation approach, modeling, and research prospects. *Biotechnol. Adv*. 35 466–489. 10.1016/j.biotechadv.2017.03.010 28351654

[B20] Li Y. Ge X. Sun Z. Zhang J. (2015). Effect of additives on adsorption and desorption behavior of xylanase on acid-insoluble lignin from corn stover and wheat straw. *Bioresour. Technol*. 186 316–320. 10.1016/j.biortech.2015.03.058 25818260

[B21] Li Y. Qi B. Luo J. Wan Y. (2016). Effect of alkali lignins with different molecular weights from alkali pretreated rice straw hydrolyzate on enzymatic hydrolysis. *Bioresour. Technol*. 200 272–278. 10.1016/j.biortech.2015.10.038 26496216

[B22] Li Z. Yao G. Wu R. Gao L. Kan Q. Liu M. (2015). Synergistic and dose-controlled regulation of cellulase gene expression in *Penicillium oxalicum*. *PLoS Genet*. 11:e1005509. 10.1371/journal.pgen.1005509 26360497 PMC4567317

[B23] Lin W. Chen D. Yong Q. Huang C. Huang S. (2019). Improving enzymatic hydrolysis of acid-pretreated bamboo residues using amphiphilic surfactant derived from dehydroabietic acid. *Bioresour. Technol*. 293:122055. 10.1016/j.biortech.2019.122055 31472409

[B24] Liu G. Zhang L. Wei X. Zou G. Qin Y. Ma L. (2013). Genomic and secretomic analyses reveal unique features of the lignocellulolytic enzyme system of *Penicillium decumbens*. *PLoS One* 8:e55185. 10.1371/journal.pone.0055185 23383313 PMC3562324

[B25] Liu Z. J. Lan T. Q. Li H. Gao X. Zhang H. (2017). Effect of bisulfite treatment on composition, structure, enzymatic hydrolysis and cellulase adsorption profiles of sugarcane bagasse. *Bioresour. Technol.* 223 27–33. 10.1016/j.biortech.2016.10.029 27771527

[B26] Lou H. Zhu J. Y. Lan T. Q. Lai H. Qiu X. (2013). pH-Induced lignin surface modification to reduce nonspecific cellulase binding and enhance enzymatic saccharification of lignocelluloses. *ChemSusChem*. 6 919–927. 10.1002/cssc.201200859 23554287

[B27] Lu X. Feng X. Li X. Zhao J. (2018). Binding and hydrolysis properties of engineered cellobiohydrolases and endoglucanases. *Bioresour. Technol*. 267 235–241. 10.1016/j.biortech.2018.06.047 30025319

[B28] Lu X. Q. Wang C. Li X. Z. Zhao J. Zhao X. B. (2017). Studying nonproductive adsorption ability and binding approach of cellobiohydrolase to lignin during bioconversion of lignocellulose. *Energy Fuels* 31 14393–14400. 10.1021/acs.energyfuels.7b02427

[B29] Lu X. Wang C. Li X. Zhao J. (2017). Temperature and pH influence adsorption of cellobiohydrolase onto lignin by changing the protein properties. *Bioresour. Technol*. 245 819–825. 10.1016/j.biortech.2017.08.139 28926914

[B30] Miao Y. Kong Y. Li P. Li G. Liu D. Shen Q. (2018). Effect of CBM1 and linker region on enzymatic properties of a novel thermostable dimeric GH10 xylanase (Xyn10A) from filamentous fungus *Aspergillus fumigatus Z5*. *AMB Express* 8:44. 10.1186/s13568-018-0576-5 29564574 PMC5862715

[B31] Miao Y. Li P. Li G. Liu D. Druzhinina I. S. Kubicek C. P. (2017). Two degradation strategies for overcoming the recalcitrance of natural lignocellulosic xylan by polysaccharides-binding GH10 and GH11 xylanases of filamentous fungi. *Environ. Microbiol*. 19 1054–1064. 10.1111/1462-2920.13614 27878934

[B32] Nakagame S. Chandra R. P. Kadla J. F. Saddler J. N. (2011). The isolation, characterization and effect of lignin isolated from steam pretreated Douglas-fir on the enzymatic hydrolysis of cellulose. *Bioresour. Technol*. 102 4507–4517. 10.1016/j.biortech.2010.12.082 21256740

[B33] Onyango M. S. Kojima Y. Aoyi O. Bernardo E. C. Matsuda H. (2004). Adsorption equilibrium modeling and solution chemistry dependence of fluoride removal from water by trivalent-cation-exchanged zeolite F-9. *J. Colloid Interface Sci*. 279 341–350. 10.1016/j.jcis.2004.06.038 15464797

[B34] Rahikainen J. L. Martin-Sampedro R. Heikkinen H. Rovio S. Marjamaa K. Tamminen T. (2013a). Inhibitory effect of lignin during cellulose bioconversion: the effect of lignin chemistry on non-productive enzyme adsorption. *Bioresour. Technol*. 133 270–278. 10.1016/j.biortech.2013.01.075 23428824

[B35] Rahikainen J. L. Moilanen U. Nurmi-Rantala S. Lappas A. Koivula A. Viikari L. (2013b). Effect of temperature on lignin-derived inhibition studied with three structurally different cellobiohydrolases. *Bioresour. Technol*. 146 118–125. 10.1016/j.biortech.2013.07.069 23920120

[B36] Rakotoarivonina H. Hermant B. Aubry N. Remond C. (2015). Engineering the hydrophobic residues of a GH11 xylanase impacts its adsorption onto lignin and its thermostability. *Enzyme. Microb. Technol*. 81 47–55. 10.1016/j.enzmictec.2015.07.009 26453471

[B37] Siddhu M. A. H. Li W. He Y. Liu G. Chen C. (2019). Steam explosion pretreatment of rice straw to improve structural carbohydrates anaerobic digestibility for biomethanation. *Environ. Sci. Pollut. Res. Int*. 26 22189–22196. 10.1007/s11356-019-05382-w 31147997

[B38] Sluiter A. Hanes B. Ruiz R. Scarlata C. Sluiter J. Templeton D. (2011). *Determination of Structural Carbohydrates and Lignin in Biomass.* Golden, CO: NREL.

[B39] Sugimoto N. Igarashi K. Wada M. Samejima M. (2012). Adsorption characteristics of fungal family 1 cellulose-binding domain from *Trichoderma reesei* cellobiohydrolase I on crystalline cellulose: negative cooperative adsorption via a steric exclusion effect. *Langmuir* 28 14323–14329. 10.1021/la302352k 22950684

[B40] Tan L. Sun W. Li X. Zhao J. Qu Y. Choo Y. M. (2015). Bisulfite pretreatment changes the structure and properties of oil palm empty fruit bunch to improve enzymatic hydrolysis and bioethanol production. *Biotechnol. J*. 10 915–925. 10.1002/biot.201400733 25866127

[B41] Tan L. Yu Y. Li X. Zhao J. Qu Y. Choo Y. M. (2013). Pretreatment of empty fruit bunch from oil palm for fuel ethanol production and proposed biorefinery process. *Bioresour. Technol*. 135 275–282. 10.1016/j.biortech.2012.10.134 23186670

[B42] Wang J. Hao X. Yang M. Qin Y. Jia L. Chu J. (2018). Impact of lignin content on alkaline-sulfite pretreatment of Hybrid Pennisetum. *Bioresour. Technol*. 267 793–796. 10.1016/j.biortech.2018.07.049 30017365

[B43] Wang Z. Zhu J. Fu Y. Qin M. Shao Z. Jiang J. (2013). Lignosulfonate-mediated cellulase adsorption: enhanced enzymatic saccharification of lignocellulose through weakening nonproductive binding to lignin. *Biotechnol. Biofuels*. 6:156. 10.1186/1754-6834-6-156 24188090 PMC3843589

[B44] Xu C. Liu F. Alam M. A. Chen H. Zhang Y. Liang C. (2020). Comparative study on the properties of lignin isolated from different pretreated sugarcane bagasse and its inhibitory effects on enzymatic hydrolysis. *Int. J. Biol. Macromol*. 146 132–140. 10.1016/j.ijbiomac.2019.12.270 31904455

[B45] Xu C. Zhang J. Zhang Y. Guo Y. Xu H. Liang C. (2019). Lignin prepared from different alkaline pretreated sugarcane bagasse and its effect on enzymatic hydrolysis. *Int. J. Biol. Macromol*. 141 484–492. 10.1016/j.ijbiomac.2019.08.263 31479677

[B46] Yang B. Wyman C. E. (2006). BSA treatment to enhance enzymatic hydrolysis of cellulose in lignin containing substrates. *Biotechnol. Bioeng*. 94 611–617. 10.1002/bit.20750 16673419

[B47] Yao L. Yang H. Yoo C. G. Meng X. Li M. Pu Y. (2017). Adsorption of cellobiohydrolases I onto lignin fractions from dilute acid pretreated *Broussonetia papyrifera*. *Bioresour. Technol*. 244 957–962. 10.1016/j.biortech.2017.08.024 28847086

[B48] Ying W. Shi Z. Yang H. Xu G. Zheng Z. Yang J. (2018). Effect of alkaline lignin modification on cellulase-lignin interactions and enzymatic saccharification yield. *Biotechnol. Biofuels.* 11:214. 10.1186/s13068-018-1217-6 30083227 PMC6069831

[B49] Yoo C. G. Li M. Meng X. Z. Pu Y. Q. Ragauskas A. J. (2017). Effects of organosolv and ammonia pretreatments on lignin properties and its inhibition for enzymatic hydrolysis. *Green Chem*. 19 2006–2016. 10.1039/C6GC03627A

[B50] Yuan Y. Zhai R. Li Y. Chen X. Jin M. (2018). Developing fast enzyme recycling strategy through elucidating enzyme adsorption kinetics on alkali and acid pretreated corn stover. *Biotechnol. Biofuels* 11:316. 10.1186/s13068-018-1315-5 30479661 PMC6245881

[B51] Zhu J. Y. Pan X. J. (2010). Woody biomass pretreatment for cellulosic ethanol production: technology and energy consumption evaluation. *Bioresour. Technol.* 101 4992–5002. 10.1016/j.biortech.2009.11.007 19969450

